# Airway closure in a patient with acute heart failure: a case report

**DOI:** 10.1186/s43044-025-00659-7

**Published:** 2025-06-20

**Authors:** Bien Huu-Thien Le, Minh Vu-Anh Phan, Khanh Bao Lieu, Yen Thi-Hai Hoang, Phuong Phan-Phuong Pham, Hau Huu-Doan

**Affiliations:** 1 Department of Critical Care Medicine, University of Medicine and Pharmacy at Ho Chi Minh City, Ho Chi Minh City, Viet Nam; 2 Intensive Care Unit, University Medical Center of Ho Chi Minh City, Ho Chi Minh City, Viet Nam; 3 SBGHC Walkerton, Toronto, Canada

**Keywords:** Case report, Airway closure, Acute heart failure, Low-flow inflation procedure, Airway opening pressure

## Abstract

**Background:**

Airway closure occurs in small airways and disconnects distal airways from proximal airways. This pathology may lead to acute pulmonary edema, worsening gas exchange and creating challenges in ventilator management.

**Case presentation:**

We report the case of a 68-year-old woman who was admitted to the hospital because of non-ST elevation acute myocardial infarction and was subsequently intubated because of refractory pulmonary edema. The low-flow inflation procedure revealed airway closure, and the positive end-expiratory pressure was titrated according to the airway opening pressure.

**Conclusions:**

Acute pulmonary edema may be accompanied by airway closure. A simple bedside procedure can indicate the presence of this complication and guide positive end-expiratory pressure setting.

## Background

Acute pulmonary edema (APE) is a life-threatening complication and the leading cause of hospitalization in patients with heart failure. The predominant injuries associated with APE are pulmonary congestion and alveolar edema, which manifest as severe dyspnea and a marked decrease in blood oxygen saturation [1]. The conventional management of APE includes ensuring oxygenation via oxygen therapies and noninvasive ventilation, diuresis, or mechanical circulatory support in some instances [2]. In addition to alveolar damage, small airway abnormalities have recently become a concern in patients with APE. Indeed, airway obstruction increases airway resistance, which can lead to cardiac asthma that manifests as inspiratory crackles and diffuse expiratory wheezing [3, 4]. At greater severity, airway injuries can cause airway closure, contributing to worsening respiratory failure and hindering the assessment of respiratory mechanics parameters and the management of mechanical ventilation.

Herein, we report a case of airway closure in a patient with acute heart failure (AHF) who was treated in the intensive care unit (ICU) at the University Medical Center of Ho Chi Minh City, together with a summary of the literature on this underrecognized issue. Written informed consent was obtained from the patient’s husband for the publication of this case report and any accompanying images.

## Case presentation

A 68-year-old woman with a clinical picture of chest pain at rest was admitted to the hospital. Her medical history was significant due to her known coronary artery disease, and she had undergone multiple stent insertions and balloon angioplasties due to recurrent stenosis. She also had a history of congestive heart failure with preserved left ventricular ejection fraction (EF), type 2 diabetes mellitus, and end-stage chronic kidney disease, for which she received intermittent hemodialysis 3 times/week.

All of her laboratory test results from the time of admission are listed in Table 1, marked by elevated troponin, N-terminal pro-B-type natriuretic peptide (NT-proBNP) and serum creatinine levels. The echocardiogram performed at admission revealed mild mitral valve regurgitation and mild aortic valve regurgitation due to degeneration, ischemic heart disease, and mildly reduced left ventricular systolic function (EF Teichholz 54%, EF Simpson 48%). She was admitted to the cardiovascular interventional department with a diagnosis of non-ST-elevation acute myocardial infarction and was prescribed nifedipine (120 mg/day), valsartan (320 mg/day), methyldopa (1500 mg/day), clopidogrel/aspirin (75/100 mg/day), atorvastatin (40 mg/day), furosemide (80 mg/day), novomix (18 units/day) and treated with intermittent hemodialysis (3 times/week).

**Table 1:** Results of laboratory tests performed at hospital admission

Test	Result	Normal range
Glucose	11.6 mmol/l	3.9–6.4 mmol/l
HbA1C	7.2%	4.4–6.0%
Urea	107.26 mg/dl	10.2–49.7 mg/dl
Creatinine	451.4 μmol/l	Female: 58–96 μmol/l
Creatinine clearance	8 ml/minute/1.73 m^2^	≥ 60 ml/minute/1.73 m^2^
Electrolytes	Sodium: 133 mmol/l Potassium: 4.1 mmol/l Chloride: 96 mmol/l Calcium: 2.4 mmol/l	136–146 mmol/l 3.4–5.1 mmol/l 98–109 mmol/l 2.1–2.5 mmol/l
Troponin T	1st: 0.242 ng/ml 2nd: 0.314 ng/ml	< 0.014 ng/ml
NT-proBNP	17,644 pg/ml	< 125 pg/ml (< 75 years old) < 450 pg/ml (≥ 75 years old)

*NT-proBNP* N-terminal pro B-type natriuretic peptide

During the next 10 days, the patient exhibited persistent angina and progressive dyspnea, requiring respiratory support beginning with nasal cannula oxygen and advancing to noninvasive ventilation (NIV). Prior to NIV, the patient underwent percutaneous coronary intervention for the first time with 4 attempts at balloon angioplasty, but the procedure was abandoned due to agitation and confusion along with an overall deterioration in her condition. The following day, her increasing dyspnea led to the use of NIV with bilevel positive airway pressure and a plan for repeat percutaneous coronary intervention. Prior to the procedure, the patient was intubated and started on volume-controlled continuous mandatory ventilation (VC-CMV). The echocardiogram revealed a reduction in left ventricular systolic function (EF of 38%, compared with that of 57% at hospital admission). During this procedure, balloon angioplasty was performed at the left main-left anterior descending I-left circumflex I artery (LM-LAD I-LCx I), and stents were placed at the LM, LM-LAD I and LAD I ostium. After the intervention, the patient’s blood oxygen saturation did not improve. As such, the patient required a high fraction of inspired oxygen (FiO_2_) and positive end-expiratory pressure (PEEP) and was subsequently transferred to the ICU.

**Fig. 1:**
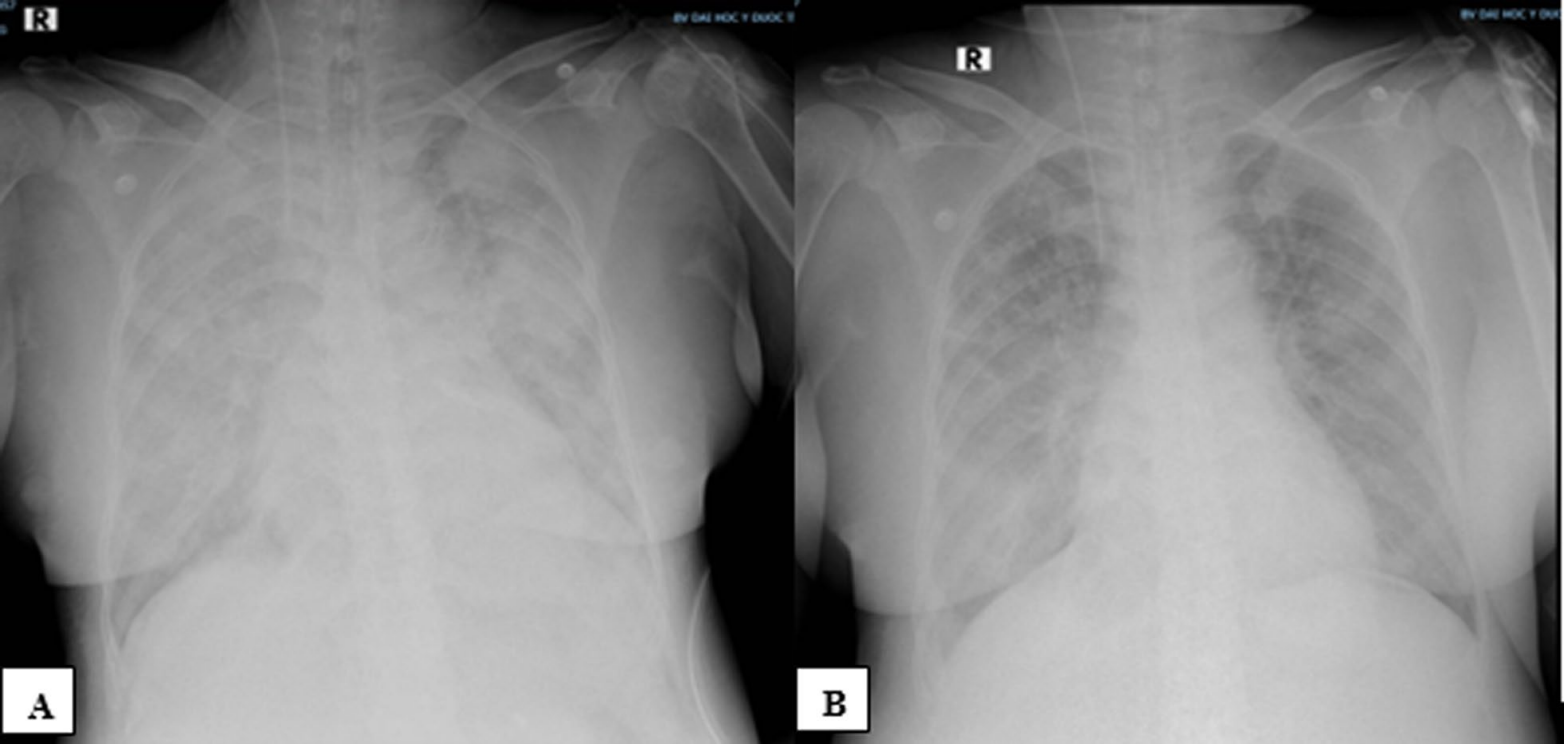
Changes in chest radiographs in the intensive care unit (ICU). **A** On the 1st day in the ICU, with worsening respiratory failure, the RALE score was 42. **B** On the 2^nd^ day, when the respiratory condition improved, the RALE score was 30

In the ICU, copious pink frothy sputum was observed in the endotracheal tube. The arterial blood gas test revealed severe hypoxemia (PaO_2_/FiO_2_ = 78), and the chest radiograph revealed bilateral perihilar opacities. The patient was diagnosed with APE due to acute decompensated chronic heart failure secondary to ischemia. The initial settings of the mechanical ventilator were as follows: the VC-CMV mode, a tidal volume of 6.9 ml/kg ideal body weight, a respiratory rate of 22 breaths/minute, an FiO_2_ of 100%, and a PEEP of 10 cmH_2_O. Midazolam/fentanyl was infused continuously. Continuous renal replacement therapy was also performed in continuous veno-venous hemodiafiltration (CVVHDF) mode, with a blood flow rate of 150–180 ml/minute, a dialysis rate of 600 ml/hour, and a replacement rate of 600 ml/hour. The patient’s condition stabilized, but by the next morning, she became agitated and started to produce pink frothy phlegm again. Her peak airway pressure was greater than the upper limit, which was set at 50 cmH_2_O, and her oxygen saturation fell rapidly without responding to increasing PEEP. Her chest X-ray taken at that time confirmed worsening pulmonary edema characterized by heterogeneous opacities and consolidations that spread throughout both lung fields (Fig. 1A).

**Fig. 2:**
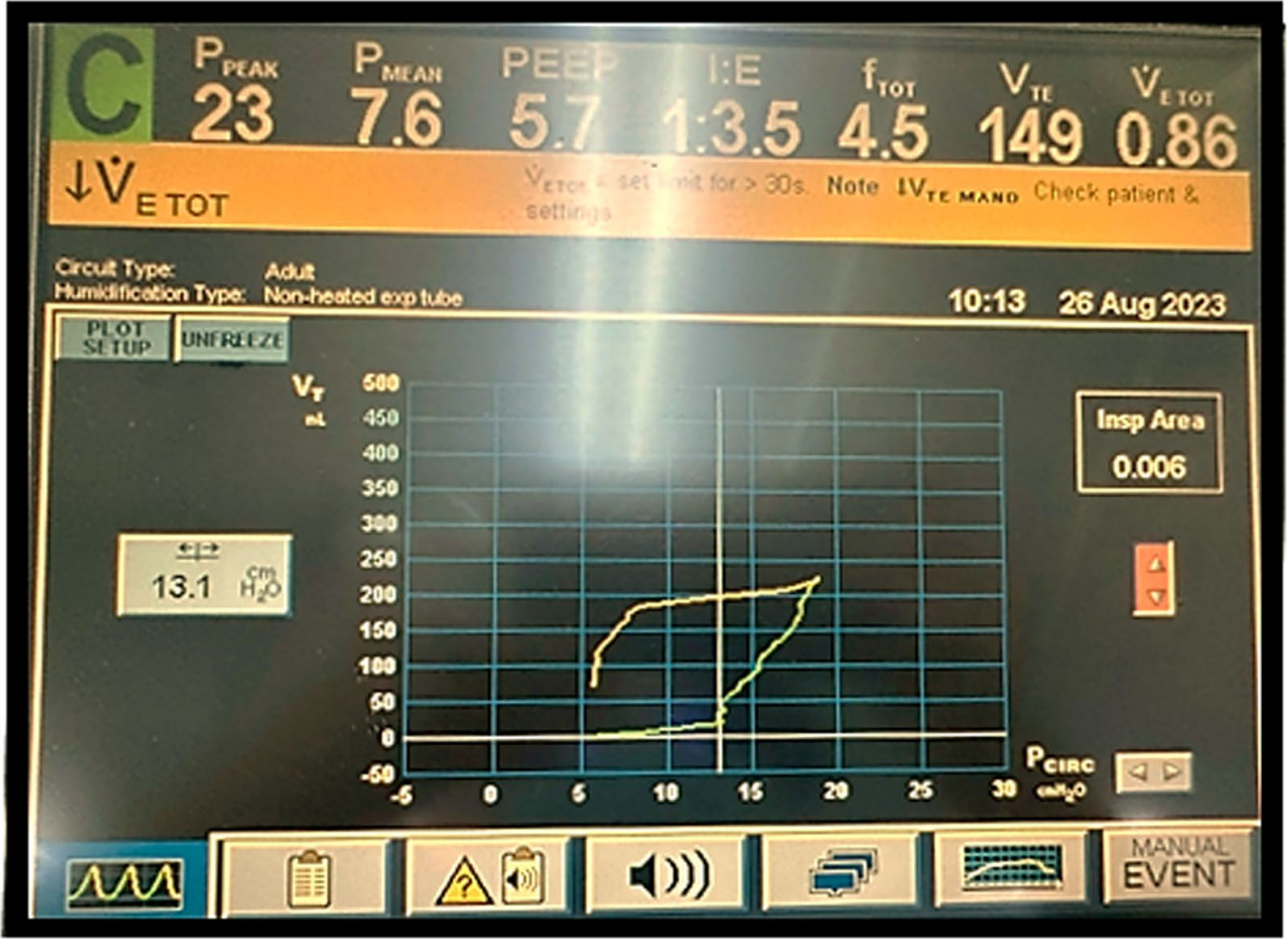
Measuring the airway opening pressure via the low-flow inflation method (with an inspiratory flow rate of 5 L/minute). On the volume‒pressure curve, before the inflection point of 13.5 cmH_2_O, the air volume inflated into the lungs was very low, approximately 25 ml, corresponding to a compliance of 1.85 ml/cmH_2_O, indicating that the airway was still closed. After the inflection point of 13.5 cmH_2_O, the inflated volume increased suddenly to over 4 ml/cmH_2_O, suggesting that the airway had opened. Compliance after the inflection point = (225–25)/(19–13.5) = 36.3 ml/cmH_2_O

Neuromuscular blockade was then used to completely abolish spontaneous respiratory efforts, and respiratory mechanics parameters were measured simultaneously (Table 2). Because our patient was suspected to have airway closure, a low-flow inflation procedure was performed to measure the airway opening pressure (AOP) (Fig. 2). With the AOP measured at 13.5 cmH_2_O, the PEEP was titrated to 14 cmH_2_O, which ultimately helped improve the patient’s blood oxygen level remarkably and concurrently helped decrease both the plateau pressure and the driving pressure. After the first day in the ICU, her fluid balance was reduced by 2070 ml, and her pulmonary edema had decreased, as shown by the chest X-ray (Fig. 1B). On the second day in the ICU, all the blood oxygen indices and respiratory mechanics parameters improved. The low-flow inflation procedure was performed again; the AOP was determined to be 9.8 cmH_2_O, and the PEEP was lowered to 10 cmH_2_O. The patient’s condition gradually improved. She was extubated after 15 days and was transferred back to the cardiovascular interventional department. She was discharged home after 47 days. Polysomnography and spirometry were performed before discharge, and no airway occlusion was revealed.

**Table 2:** Respiratory conditions, respiratory mechanics, and mechanical ventilator parameters measured in the ICU

Parameter	At ICU admission	1st day, morning	1st day, evening	2nd day, morning
Arterial blood gas pH PaCO_2_ (mmHg) PaO_2_ (mmHg) PaO_2_/FiO_2_	7.43 36.0 78.0 78.0	7.35 35.3 94.4 94.4	7.42 34.6 76.1 126.9	7.39 38.2 119.0 264.4
FiO_2_ (%)	100	100	60	45
Respiratory rate (breaths/minute)	24	22	22	20
VT (ml/kg)	6.9	7.3	8.1	8.1
PEEP (cmH_2_O)	8	10	14	10
Peak inspiratory pressure (cmH_2_O)	NA	48	35	28
Plateau pressure (cmH_2_O)	NA	35	30	22
Driving pressure (cmH_2_O)	NA	25	16	12
Airway opening pressure (cmH_2_O)	NA	13.1	13.5	9.8
Compliance (ml/cmH_2_O)	NA	17.3	17.5	23.3
Modified compliance (ml/cmH_2_O)	NA	21.8	16.9	22.9

*ICU* intensive care unit, *NA* not available, *PEEP* positive end-expiratory pressure, *VT* tidal volume

## Discussion

This clinical case demonstrates that in patients with APE, in addition to alveolar damage, airway closure may play an important role in causing gas exchange disturbances and challenges in ventilator management. Airway closure, in which small airways completely collapse, disconnects distal airways from proximal airways. More commonly, airway closure is recognized in patients with airway diseases such as asthma or chronic obstructive pulmonary disease and has also recently been reported in patients with acute respiratory distress syndrome (ARDS) [5].

There are several methods used to evaluate patients with small airway diseases, including direct (endobronchial optical coherence tomography, multidetector computerized tomography (CT), and hyperpolarized gas magnetic resonance imaging (MRI)) and indirect (plethysmography, oscillometry, and inert gas washout) methods [6]. However, these methods cannot be applied clinically in patients undergoing invasive mechanical ventilation. In the present case, we used a low-flow inflation procedure to abolish the effect of resistance on airway pressure when respiratory system compliance was estimated. On the volume‒pressure curve, before the inflection point of 13.5 cmH_2_O, the compliance of the respiratory system was very low (approximately 1.85 ml/cmH_2_O), which was comparable to the respiratory system compliance of patients with APE undergoing invasive ventilation reported in the LUNG SAFE study [7]. In fact, this very low compliance corresponded to the compliance of the ventilator circuit measured in vitro (2.38 ml/cmH_2_O), so we speculated that the patient’s airways were still closed. After the inflection point of 13.5 cmH_2_O, the volume‒pressure curve increased dramatically, with a calculated compliance of approximately 36 ml/cmH_2_O, which was proportional to the compliance of the patients with severe APE in the LUNG SAFE study. This inflection point on the volume‒pressure curve could be presumed to be the lower point of lung recruitment in patients with acute lung injury or atelectasis. Although no CT image was available to exclude atelectasis, compliance lower than 2 ml/cmH_2_O never corresponds with lung parenchymal damage, even in the most severe cases. Thus, we believe that this patient experienced airway closure at an AOP of 13.5 cmH_2_O.

There are several causes of airway closure in patients with AHF, including intraluminal reasons, such as a reduction in or dysfunction of surfactant or the accumulation of fluid, which results in the formation of bubbles that completely block bronchioles [1], and extraluminal reasons, such as increased interstitial density around bronchioles due to congestion [4]. Because we were unable to measure extravascular lung water to objectively evaluate pulmonary congestion, we used the radiographic assessment of lung edema (RALE) score because of its correlation with pulmonary congestion [8] and noted that when pulmonary congestion improved, the AOP also decreased. It is therefore plausible that pulmonary congestion plays a fundamental role in the development of airway closure in heart failure patients. Indeed, airway diseases have been reported in patients with heart failure in many previous studies [9, 10]. In one such study, patients with systolic heart failure underwent pulmonary function testing twice, once prior to discharge and again after 6 months, and their airway obstruction indices, such as the forced expiratory volume in one second (FEV1) and the FEV1/FVC ratio (forced vital capacity: FVC), improved only slightly or did not improve, whereas the parameters indicative of lung volume, including the FVC or total lung capacity (TLC), improved significantly [9]. A smaller cohort of patients with AHF without a history of chronic obstructive pulmonary disease were also shown to have both restrictive and obstructive ventilatory dysfunction at admission [10]. After treatment for 4 weeks, the FEV1 changed only marginally, but the FVC and TLC improved markedly. In addition, the difference between the TLC measured via spirometry and the nitric oxide diffusing capacity decreased from 1.04 to 0.52 L. These studies revealed that airway closure leads to a reduction in lung volume in patients with AHF, which, if recognized and measured, can improve with appropriate treatment.

Absolute airway closure can cause lung volume reduction and gas exchange dysfunction. When airways close partially, the terminal respiratory units tend to open and close repeatedly, leading to ventilator-induced lung injury [11]. Partial airway closure can also increase respiratory failure risk and breathing effort, which leads to the aggravation of pulmonary edema, as observed in this patient on the first day in the ICU. In terms of calculating respiratory mechanics indices, missing AOP measurements may result in inaccurate measurements of respiratory system compliance or lung recruitability.

In the absence of any guidelines for managing airway closure in patients with AHF, the PEEP was set just above the AOP, as recommended in the guidelines for patients with ARDS who experience this phenomenon [11]. This PEEP level helps keep the airway open and enhances gas exchange without exaggerating air trapping and intrinsic PEEP. However, in terms of the underlying cause of disease, only measures to treat heart failure and decrease pulmonary congestion can resolve airway closure completely.

## Limitations

Our patient recovered with appropriate respiratory support and aggressive decongestion, but we recognize that limitations in our diagnostic ability remain. First, we did not have any imaging methods to directly evaluate the airway closing phenomenon. We indirectly measured the change in pulmonary compliance on the basis of the ventilator volume–pressure curve. Second, we could not quantify the alveolar surfactant concentration or the volume of extravascular lung water. We only assessed the patient’s pulmonary congestion via the RALE score and were thus unable to accurately define the mechanism of airway closure. Finally, we could not perform body plethysmography, which is the best way to evaluate TLC.

## Conclusion

In addition to causing alveolar damage, APE can be accompanied by airway injury, which can lead to airway closure. If not detected, airway closure may worsen respiratory failure and ventilator-induced lung injury, leading to many difficulties in ventilator management. This complication can be recognized easily via the low-flow inflation technique. To keep the airways opened, a PEEP level higher than the AOP might be necessary. Nevertheless, because airway closure is a dynamic phenomenon, the AOP needs to be regularly measured to adjust the PEEP appropriately.

## Data Availability

No datasets were generated or analysed during the current study.

## Abbreviations

AHF: Acute heart failure
AOP: Airway opening pressure
APE: Acute pulmonary edema
ARDS: Acute respiratory distress syndrome
CT: Computerized tomography
CVVHDF: Continuous veno-venous hemodiafiltration
EF: Ejection fraction
FEV1: Forced expiratory volume in one second
FiO_2_: Fraction of inspired oxygen
FVC: Forced vital capacity
ICU: Intensive care unit
LM—LAD I—LCx I: Left main-left anterior descending I-left circumflex I artery
MRI: Magnetic resonance imaging
NIV: Noninvasive ventilation
PEEP: Positive end-expiratory pressure
TLC: Total lung capacity
VC-CMV: Mechanical ventilator with volume control mode
